# Implementation and evaluation of a community-based mindful walking randomized controlled trial to sustain cognitive health in older African Americans at risk for dementia

**DOI:** 10.1186/s12877-024-05090-2

**Published:** 2024-07-04

**Authors:** Chih-Hsiang Yang, Jongwon Lee, Sara Wilcox, A. Caroline Rudisill, Daniela B. Friedman, Jonathan G. Hakun, Jean Neils-Strunjas, Jingkai Wei, Margaret C. Miller, Megan D. Byers

**Affiliations:** 1 Department of Exercise Science, Arnold School of Public Health, University of South Carolina, Columbia, SC USA; 2 Department of Health Promotion, Education, and Behavior, Arnold School of Public Health, University of South Carolina, Columbia, SC USA; 3 Department of Epidemiology and Biostatistics, Arnold School of Public Health, University of South Carolina, Columbia, SC USA; 4 Department of Communication Sciences and Disorders, Arnold School of Public Health, University of South Carolina, Columbia, SC USA; 5 Prevention Research Center, Arnold School of Public Health, University of South Carolina, Columbia, SC USA; 6 Office for the Study of Aging, Arnold School of Public Health, University of South Carolina, Columbia, SC USA; 7 Department of Neurology, The Pennsylvania State University, College of Medicine, Hershey, PA 17033 USA; 8 Department of Psychology, The Pennsylvania State University, University Park, PA 16802 USA; 9 Department of Public Health Sciences, The Pennsylvania State University, College of Medicine, Hershey, PA 17033 USA

**Keywords:** Lifestyle physical activity, Mind-body intervention, Alzheimer’s disease and related dementias, Cognitive health, Prevention research

## Abstract

**Background:**

With an increasing proportion of older adults and the associated risk of Alzheimer’s Disease and Related Dementias (ADRD) around the globe, there is an urgent need to engage in ADRD risk reduction efforts. African American (AA) older adults in the U.S. are disproportionally impacted by ADRD compared to other races and ethnicities. Mindful walking integrates two potentially protective factors of ADRD by elevating mindfulness and physical activity (i.e., walking), resulting in a synergistic behavioral strategy that is feasible and safe for older adults. However, the efficacy of applying this intervention for cognitive health outcomes has not been evaluated using experimental designs.

**Methods:**

This paper documents the goal and protocol of a community-based, mindful walking randomized controlled trial to examine the short- and longer-term efficacy on cognitive and other health-related outcomes in ADRD at-risk AA older adults. The study outcomes include various brain health determinants, including cognitive function, quality of life, psychological well-being, physical activity, mindfulness, sleep, and overall health status. In addition, the estimated costs of program implementation are also collected throughout the study period. This study will recruit 114 older adults (ages 60+ years) with elevated ADRD risk from the Midlands region of South Carolina. Older adults are randomly assigned to participate in 24 sessions of outdoor mindful walking over three months or a delayed mindful walking group (*n*=57 in each group). Participants in both groups follow identical measurement protocols at baseline, after 12 weeks, after 18 weeks, and after 24 weeks from baseline. The outcome measures are administered in the lab and in everyday settings. Costs per participant are calculated using micro-costing methods. The eliciting participant costs for mindful walking engagement with expected results are reported using the payer and the societal perspectives.

**Discussion:**

This study will generate evidence regarding the efficacy of mindful walking on sustaining cognitive health in vulnerable older adults. The results can inform future large-scale effectiveness trials to support our study findings. If successful, this mindful walking program can be scaled up as a low-cost and viable lifestyle strategy to promote healthy cognitive aging in diverse older adult populations, including those at greatest risk.

**Trial registration:**

ClinicalTrials.gov number NCT06085196 (retrospectively registered on 10/08/2023).

**Supplementary Information:**

The online version contains supplementary material available at 10.1186/s12877-024-05090-2.

## Background

Alzheimer’s disease and related dementias (ADRD) have become one of the leading causes of death and disability [1–3]. There is currently no effective treatment to cure ADRD. Over six million Americans and one in every nine older adults are currently living with ADRD, and the projected total ADRD-related health costs will rise to one trillion a year by 2050 [4]. Among individuals living with ADRD, African Americans (AA) are over-represented compared to other race and ethnicity groups [5]. AA older adults (ages 65+) have the highest ADRD prevalence rate (~ 14%) compared to other racial and ethnic groups [6, 7]. Thus, ADRD has placed a tremendous economic burden on AA and their families, who bear 33% of total ADRD costs nationally [8]. Additionally, as new pharmacological treatments become available, there is concern that AAs, among other populations, may not be able to afford the cost [9]. Most ADRD prevention programs to date are overwhelmingly conducted with non-Hispanic White individuals, limiting our understanding of preventing ADRD in the AA population [10, 11]. This study commits to sustaining cognitive health among AA older adults by developing and implementing a mindful walking intervention to serve this priority population urgently requiring preventive strategies to reduce ADRD risk.

The Advisory Council on Alzheimer’s Research, Care, and Services and the Physical Activity Guidelines Advisory Committee identified physical activity (especially moderate-to-high intensity) as a major protective factor for brain health [12, 13]. Existing older adult physical activity programs usually require moderate-to-high mobility levels to enter the programs [14–16]. However, approximately 28% of older adults have mobility barriers, limiting their ability to exercise at the recommended intensity levels to reap the benefits [17]. Older adults with higher ADRD risk also experience more physical barriers or mobility limitations [18], making them likely to fail inclusion criteria for exercise programs or preventive clinical trials. This study focuses on a relatively achievable physical activity type (walking) with the incorporation of mindfulness practice to sustain brain health in ADRD at-risk older AAs.

Research suggests that accruing physical activity at a lower intensity, such as walking, may improve cognition and related brain structures in older adults [19–21]. Walking is an ideal intervention target as it is the primary daily activity and the most familiar physical activity type among older adults [22]. It is also considered safe and appealing to most older adults [23–25]. Several older adult walking programs have been successfully applied in shopping malls [16, 26]. However, these mall walking programs are usually restricted to the non-business time windows for walkers, and the group walking setting provides less flexibility for older adults who may not meet the group walking schedule. The current mindful walking program is implemented in an open space accessible at any time for older adults in the community. It also does not require a walking group or walking partners, which mitigates one of the barriers for older adults with limited social networks or who prefer to engage in physical activity alone [23]. Moreover, there is evidence to suggest that outdoor walking or physical activity confers more health benefits than indoor walking [27, 28].

When walking at a slower pace, it is easy to incorporate mindfulness practice, which is another promising strategy for sustaining brain health in older adults [29–32]. Available literature suggests that mindfulness programs appeal to older adults, as indicated by the high program compliance and completion rates [29]. Mindfulness practice cultivates individuals’ attention and awareness in every present moment and engages individuals’ present-moment experience in a non-judgmental manner [33, 34]. While existing mindfulness-based interventions primarily focus on mental health outcomes (e.g., stress reduction) [35–37], the neurobiological mechanisms underlying mindfulness practice are also closely linked to cognitive ability [38–40]. When practicing mindfulness, various brain regions that are linked to sustained attention and executive function are stimulated (e.g., anterior cingulate cortex, autonomic nervous system) [31, 32, 38, 41, 42]. Based on the findings that walking and mindfulness practice are both promising strategies to sustain cognitive function and well-being in older adults, combining both strategies may yield a synergy to benefit brain health [43–45].

Our recent work suggests that short bouts of mindful walking sessions are feasible and safe to implement in the community and may elevate older adults’ cognitive function in the short term [46, 47]. However, the intervention efficacy of mindful walking on sustaining cognition has not been tested using an experimental approach, especially among ADRD at-risk older populations. Therefore, this study is designed to narrow the literature gap using a randomized controlled trial (RCT) to examine the potential cognitive benefits generated from mindful walking engagement.

## The current study

The primary goal of this study is to evaluate the short- and longer-term effects of mindful walking intervention on cognitive health outcomes in AA older adults at risk for ADRD. This mindful walking program partners with local organizations, federal agencies, and stakeholders to recruit and retain participants from our priority population. Our team administers lab- and device-based measures on multiple occasions to evaluate the intervention effect across 24 weeks. In addition, this study estimates the total cost of planning and implementing the current mindful walking program. Finally, we evaluated program recruitment and retention for future large-scale implementation studies. The Institutional Review Board approved the study protocol and all materials described in this paper (Pro00123487).

## Methods

**Fig. 1:**
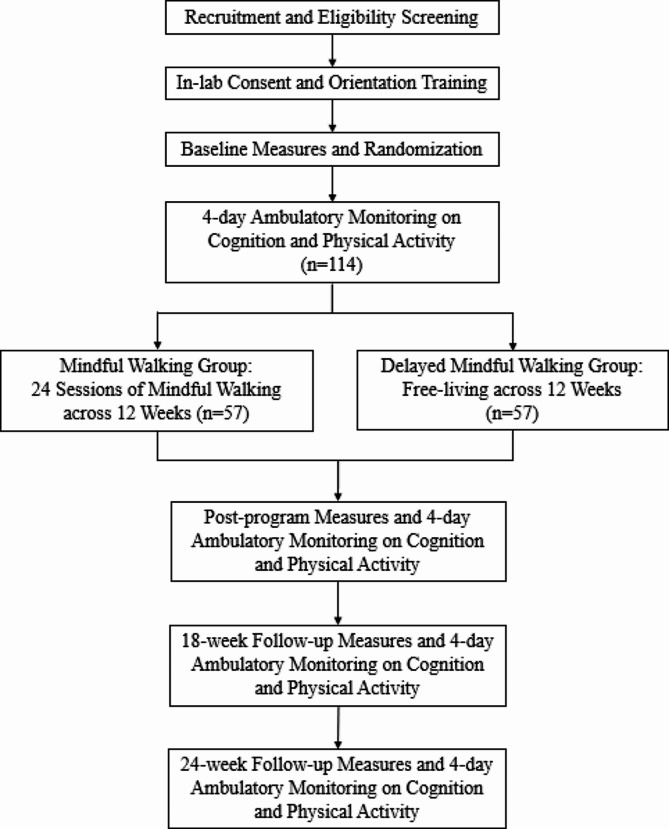
Overview of study design

This study is a two-arm, randomized controlled trial (RCT) comparing an outdoor mindful walking intervention versus a delayed intervention comparator. Figure 1 depicts the study design and measurements throughout the trial. This program is implemented in South Carolina (SC), which has a pronounced ADRD race disparity. Based on the SC ADRD registry, AAs comprise 27% of the SC residents diagnosed with ADRD. In addition, older SC AA residents (ages 65+) are 35% more likely to have ADRD compared to non-Hispanic White older adults living in SC [48]. This mindful walking study took place at the South Carolina State House outdoor walking trail located in downtown Columbia.

Older adults’ cognitive function is the primary study outcome in this study. The secondary outcomes are quality of life, overall health status, perceived stress, depressive symptoms, physical activity (steps), sleep, and mindfulness. The short-term effect refers to any differences in the primary and secondary outcomes from baseline to post intervention (after the 12 weeks) between the two groups. The longer-term effect refers to differences in the primary and secondary outcomes between baseline and 18 weeks as well as 24 weeks between the two groups. More detailed descriptions in the primary and secondary study outcomes are provided in the Measures section. We hypothesize that the short-term and the longer-term differences of the primary and secondary outcomes can be observed between the two groups after the intervention period.

### Participants

This study will recruit a total of 114 gender-inclusive older AA adults aged 60 and above in the Midlands region of SC. Instead of using the typical age 65 cutoff for older adults, the age of 60 is selected because this study is designed to inform risk-reductive behavioral strategies to sustain cognitive health during the early preclinical stage of ADRD. Our inclusion and exclusion criteria, as outlined below, also seek to make this program available to most AA older adults who have increased risks for developing ADRD (e.g., does not require prominent levels of physical capacity and mobility), and thus, may benefit from participating in mindful walking.

The inclusion criteria included: (1) African American older adults age 60 and above, (2) have at least one ADRD risk factor (including experiencing subjective cognitive decline or memory complaints, having a family history of ADRD, being physically inactive at enrollment based on the 2018 Physical Activity Guidelines for Americans, having higher BMI (≥ 25)), (3) have adequate hearing and visual ability to complete study tasks and assessments, (4) English proficiency, (5) be medically stable with or without medication, (6) be able to provide informed consent, and (7) be willing to be randomized to one of the two groups. The exclusion criteria included: (1) have a clinical diagnosis of ADRD or other brain abnormalities (e.g., strokes, epilepsy, Parkinson’s disease), (2) have a clinical diagnosis of a psychiatric disorder (i.e., major depression, post-traumatic stress disorder, bipolar disorder), (3) unable to walk independently (i.e., need caregiver’s assist), (4) plan to have surgery or relocate outside the area within the next 12 months, and (5) currently participate in other study involving physical activity, mindfulness or meditation, or cognitive training. We selected these exclusion criteria to ensure that older adults have the capacity to carry out the study protocol and that their current health condition will not bias or interfere with the study outcomes.

### Recruitment

This study engaged with community partners who share the same mission to promote healthy aging and address ADRD in older populations (e.g., SC AARP, SC Alzheimer’s Association Chapter, SC Department on Aging). These local partners have rich experience interacting with underrepresented racial and ethnic groups, so they are assisting in recruiting our priority population of AA participants. They also serve on our community advisory board (CAB) to provide insights and feedback on the planning, outreach, recruitment, and implementation of the mindful walking program.

In addition to the community-engaged recruitment approach, we also leverage the SC Alzheimer’s Disease Registry, the Dementia Dialogues® Certified Instructors in SC, and the Office for the Study of Aging’s (OSA) listserv, all hosted by the OSA at the University of South Carolina. The ADRD registry is the most comprehensive database among the only three population-based registries in the US [48]. Our study does not recruit older adults with cognitive impairment, but this unique platform can help us identify eligible AA older adults by sending out promotional materials/mailers to the homes of individuals with ADRD to inform eligible family members and caregivers. Normally, the family members or caregiver(s) of the care recipient in the ADRD registry monitor the mails and they likely meet the inclusion criteria to participate in this study. Other general recruitment methods are also carried out by the research team, including distributing fliers and mailers at community events, senior living facilities, local churches, and retirement centers. We also send out electronic flyers to relevant email listservs, online forums/support groups, and paid public service announcements. The study recruitment has occurred on a rolling basis and will continue until May 2024.

#### Screening and First Lab visit

Potential participants are contacted by the study staff to complete a phone screening to determine their eligibility based on the inclusion/exclusion criteria. Eligible older adults who pass the screening are invited for the first in-person baseline lab visit. During this lab visit (Week 0), a trained staff provides a brief study description to participants and obtains their informed consent. After giving consent, participants are guided to complete anthropometric and baseline measures regarding their demographics, mental and physical health status, and other individual characteristics (see Measures section). Lastly, the staff demonstrates to the participants how to wear the activity monitor and completing smartphone tasks that will be performed throughout the study period. After the demonstration, participants practice the tasks on the smartphone and wear the activity monitor to ensure they feel comfortable using these technologies. When participants exit the first visit, they receive a study handbook that includes detailed instructions regarding the study protocols and study contact information.

#### Randomization

After completing the baseline measures, participants are randomly assigned to the mindful walking group, or the delayed mindful walking group based on block randomization by the staff. Random blocks were generated by a methodologist on the team, stratified by sex (male vs. female) and baseline cognitive function (normal cognition vs. mild cognitive decline based on MoCA; see Measures section) to balance the two groups. The rest of the research team is blinded to the condition allocation sequence kept in sealed envelopes. Participants assigned to the intervention group schedule their 24 mindful walking sessions with the staff from week 1 to week 12. Participants assigned to the delayed mindful walking group are asked to live their normal lives and follow their regular daily routines without changing their behaviors until they start their mindful walking sessions after week 24. The measurement schedule is identical between the two groups from baseline to the last follow-up to compare group differences with or without the intervention.

#### Outdoor mindful walking sessions

The mindful walking group participated in 24 sessions of outdoor mindful walking supervised by trained research staff over 12 weeks. Participants schedule two walking sessions on two different days per week, with at least one day apart between two consecutive sessions. Each session involves 30 min of individual walking along a flat, designated oval walking trail (~ 0.4 miles for one lap) in a green space surrounding the South Carolina State House. The mindful walking program is designed as an individual activity to facilitate the elevation of a mindfulness state without distraction from social interactions.

On every scheduled mindful walking day, participants meet with the staff at the walking site between 8 am and 6 pm. The research staff greet participants and instruct them on the specific mindful walking skill for that day’s walking through a brief demonstration. During participants’ mindful walking, the staff provides supervision from a short distance (around the central spot of the oval walking trail) and monitors their walking time. Each participant also completes a brief survey on a smartphone right before and after their 30-minute walk. Lastly, the staff conducts a brief interview after participants finish the post-walking survey to understand their experiences during mindful walking. Participants are encouraged to select their most relaxing and comfortable way of walking at a relatively slower pace compared to their typical walking. Walking at a slower and more comfortable speed can help elicit a state of mindfulness and elevate their awareness of present-moment experiences [49], which is a critical component of our intervention. Walking sessions can be rescheduled with participants in case of adverse weather conditions (e.g., storms, heavy rain, hot temperatures) or unexpected incidents (e.g., family emergency).

Across the 24 sessions, the trained research staff instruct participants to practice three fundamental mindful walking skills. These skills involve being aware and attentive to the rhythm of their breathing, being aware and attentive to the movement of each step, and mentally scanning the whole body top-down and bottom-up to identify and accept sensations or feelings that arise in every present moment [33, 50]. These three basic mindful walking skills are introduced and conducted progressively as the walking sessions proceed (see mindful walking session design in Table 1). While various mindfulness skills currently exist in interventions to promote health, we focused on these fundamental mindfulness practices because they are easy for the general population to follow without any prior experience. Thus, this program can enhance participant reach and acceptability.

**Table 1:** Components of the 24 mindful walking sessions

Walking Session	1	2	3–4	5–6	7–8	9–10	11–12	13–14	15–16	17–18	19–20	21–22	23–24
Practice Mindful skill 1	-	X	X	X	-	-	X	X	-	-	X	X	X
Practice Mindful skill 2	-	-	-	-	X	X	X	X	-	-	X	X	X
Practice Mindful skill 3	-	-	-	-	-	-	-	-	X	X	X	X	X

Note: Each walking session lasts for 30 min. Mindfulness skill 1: practice walking while sustaining attention to the rhythm of every breath. Mindfulness skill 2: practice awareness of the gentle heel-to-toe rhythm for each step. Mindfulness skill 3: practice doing a mental body scan to expand attention to any bodily sensations or feelings that arise during walking. The first walking session does not involve mindful walking skill. It is designed to provide a quick introduction to the walking trail, familiarize participants with the walking route, and practice walking at a comfortable pace. The 23rd and 24th walking sessions overlap with the 14-day monitoring period of physical activity (using activPAL) and everyday cognition (using the M2C2 app)

#### Safety considerations

Our mindful walking intervention is considered safe for older adults because it only requires participants to perform a mental activity in addition to walking (i.e., a lifestyle behavior they engage in daily life). There were also no adverse events reported in our previous mindful walking feasibility study [46]. However, we implement extra measures and protocols to ensure participant safety during the intervention period. Prior to each walking session, the research staff conducts a safety check of the walking trail. When participants are walking, the staff observes them from a distance (within approximately 200 feet) to respond promptly to any incident. The research staff also makes weekly check-in phone calls to ask participants if they experienced any physical or psychological discomforts or adverse events relevant to mindful walking. In the event of any moderate or severe symptoms or the development of health conditions, the research team will obtain necessary information to determine the severity and relatedness to the intervention. If appropriate, the team will encourage participants to see a healthcare provider and will report the adverse event(s) to the IRB and the relevant agencies.

### Follow-up lab visits

At the post-intervention visit after completing the last mindful walking session at week 12, participants in both groups meet with the research staff to complete in-lab assessments that are identical to the baseline measures (except the demographics surveys). Participants in both groups also meet with the research staff for two follow-up in-lab assessments after 18 and 24 weeks to evaluate the longer-term intervention effects. On the last visit, participants can provide their feedback on the study, and they will receive monetary compensation based on their compliance (up to $200).

### Measures

Participants in both groups completed identical lab-based measures at four time points (baseline, after 12 weeks, after 18 weeks, and after 24 weeks). They also conducted four measurement bursts (repeated assessments delivered multiple times per day for several days) of ecological momentary assessment (EMA) right after the four lab visits to understand how the study outcomes differ between the groups in real-life settings in short- and longer- terms. Trained research staff instruct participants on completing these in-lab and EMA measures during the lab visits. The measures and their administration timing are summarized in Table 2.

**Table 2:** Summary of study outcomes, measurement occasions, and validated tools

Measures	Format	Administration Timing	Tools
**Primary Outcomes**			
Cognitive function	In lab	Baseline, 12,18,24 weeks	MoCA, CERAD
	Mobile	Baseline, 19, 25 weeks (4 days each)	M2C2 Mobile application
		Weeks 12 and 13 (14 days)	
**Secondary Outcomes**			
Quality of life	In lab	Baseline, 12,18,24 weeks	EQ-5D-5 L
Physical activity	In lab	Baseline, 12,18,24 weeks	CHAMPS
	Mobile	Weeks 12 and 13 (14 days)	ActivPAL accelerometer
Mindfulness	In lab	Baseline, 12,18,24 weeks	FFMQ, MAAS
Overall health status	In lab	Baseline, 12,18,24 weeks	SF-36
Mobility and gait speed	In lab	Baseline, 12,18,24 weeks	10-meter walk test
Perceived stress	In lab	Baseline, 12,18,24 weeks	PSS-10
Depressive symptoms	In lab	Baseline, 12,18,24 weeks	CES-D
Sleep quality	In lab	Baseline, 12,18,24 weeks	PSQI
**Other Covariates**			
Demographics	In lab	Baseline	Survey questions
Height and weight	In lab	Baseline	PhenX Toolkit
Body Mass Index (BMI)	In lab	Baseline	Calculated (= kg/m^2^)
Waist circumference	In lab	Baseline	PhenX Toolkit
Blood pressure	In lab	Baseline	PhenX Toolkit
Health literacy	In lab	Baseline	Validated survey item
Grip strength	In lab	Baseline	PhenX Toolkit
Daily functioning	In lab	Baseline	IADL
Social support	In lab	Baseline	PSSS
Medication use	In lab	Baseline	Survey questions

Note: MoCA: Montreal Cognitive Assessment; CERAD: Consortium to Establish a Registry for Alzheimer’s Disease subjective cognitive decline scale; M2C2: Mobile Monitoring of Cognitive Change app; EQ-5D-5L: EuroQol 5 dimensions and 5 levels of response quality of life survey; CHAMPS: Community Health Activities Model Program for Seniors physical activity questionnaire; FFMQ: Five Facet Mindfulness Questionnaire; MAAS: Mindfulness Attention Awareness Scale; SF-36: 36-Item Short Form Health Survey; PSS-10: 10-item Perceived Stress Scale; CES-D: Center for Epidemiological Studies-Depression Scale; PSQI: Pittsburgh Sleep Quality Index; IADL: Instrumental Activities of Daily Living Scale; PSSS: Perceived Social Support Scale

### Demographics, Anthropometrics, and individual characteristics

Participants self-report their demographics (age, sex, race, education level, household composition, and marital status) during the baseline lab visit. The research staff measures participants’ height and weight (for BMI calculation), waist circumference, blood pressure, and grip strength in duplicate using the PhenX Toolkit protocol in all lab visits [51]. In addition, four validated self-report measures are included in each lab visit, including: (1) Instrumental Activities of Daily Living Scale (IADL) measures participants’ independence and functioning in daily activities [52], (2) Perceived Social Support Scale (PSSS) measures participants’ perceived social support [53], (3). the types of medications currently taken, comorbidities (Charlson et al., 1994), and (4) and health literacy using one valid item [54]. All self-report data are collected via the Qualtrics online survey platform. These baseline measures serve as covariates in our analysis to adjust for individual differences at enrollment that may contribute to the study outcomes.

### Primary outcome: Multimodal measures of Cognition

The current literature has not established the specific cognitive domains that are most responsive to mindful walking interventions. Thus, this study applied both in-lab and mobile-based cognitive measures (performance-based and subjective ratings) to broadly assess cognitive health outcomes and compare results between different modes of measures. This approach provided more elaborate data regarding which cognitive domains may be more sensitive to mindful walking engagement. If positive results are observed across all cognitive measures, it will also demonstrate convergent evidence of mindful walking for sustaining cognitive health broadly [55].

***In-lab cognitive assessment***. During each lab visit, a certified research staff administers the Montreal Cognitive Assessment (MoCA) and the subjective cognitive decline scale from the Consortium to Establish a Registry for Alzheimer’s Disease (CERAD) to assess participants’ cognitive function. The MoCA is a sensitive neuropsychological measure for differentiating individuals with normal cognitive aging from those with mild cognitive decline [56]. It aggregates subscales from different cognitive domains (visuospatial/executive function, naming, memory, attention, language, abstraction, delayed recall, orientation) with a total maximum score of 30. A cutoff score of 26 on MoCA at baseline is used for the block randomization to ensure participants are comparable in baseline cognition between the two groups [56]. The alternate forms of MoCA (v8.1 to v8.3) are applied for each measurement occasion to minimize potential learning effects [57]. The CERAD cognitive decline scale is also a valid tool that can detect the occurrence of early cognitive deficits before other cognitive tests can capture them during the ADRD preclinical stage [58, 59].

***Mobile cognitive assessment.*** This study applies ultra-brief smartphone-based cognitive tests from the NIH-funded M2C2 (Mobile Monitoring of Cognitive Change) application platform to measure participants’ cognitive function in naturalistic settings during four intensive measurement bursts [60, 61]. The first burst lasts for four days after the initial lab visit to establish participants’ baseline everyday cognitive function. The second burst is scheduled for 14 days from weeks 12 to 13. The weeks 12 measures overlap with the last two sessions of mindful walking, reflecting the cognitive function changes during the intervention period for the intervention group. We include the week-12 measures to understand whether participants’ cognitive function differs with and without the mindful walking intervention exposure (as a secondary analysis). However, the short-term efficacy of everyday cognition will be evaluated using the data collected in weeks 13 after the intervention group completes all the walking sessions. The third and the fourth bursts are scheduled right after the 18-week and after the 24-week lab visits, respectively. These longer-term measurement bursts will determine if there are sustained intervention effects.

During each measurement burst, the M2C2 app delivers four semi-random daily notifications during waking hours. Upon hearing a notification, participants use the smartphone touch screen to complete three ultra-brief cognitive tests in everyday contexts. The three brief cognitive tests include the symbol search (targeting mental processing speed), the grid memory (targeting visuospatial working memory), and the color shapes (targeting executive function). These validated cognitive tests are selected due to their sensitivity to the cognitive domains influenced by lifestyle behaviors, cognitive aging, and age-related neuropathology [62, 63]. Participants are asked to ignore prompts delivered during an incompatible activity (e.g., driving, cooking, biking) for safety purposes.

In the Symbol Search test, participants select a combination of symbols from the bottom of the screen that matched the one presented at the top of the screen as fast as they can. In the Grid Memory task, participants need to recall the locations of three red dots that appeared during the previous brief study phase. A distraction page (a letter cancellation task) is embedded between the study and the test phase. In the Color Shapes test, participants determine whether the combination of colors and shapes is identical among three visual objects (distributed throughout the array) between the study and test arrays. This cognitive test is highly sensitive to early subtle cognitive changes in the presence of ADRD pathology prior to formal detection from standard neuropsychological tests [64]. Each day, participants encounter different test stimuli for each brief cognitive test at each EMA notification.

The primary outcome of the Symbol Search test is the median response time (RT) for the accurate trials; the primary outcome of the Grid Memory test is the mean error distance of the dots between the study and test phases; the primary outcome of the Color Shapes test is the corrected recognition rate (i.e., the hitrate minus the false alarmrate). In our pilot testing in older adults, participants can complete the three ultra-brief cognitive tests within a total of 5 min (18 trials for Symbol Search, 3 trials for Grid Memory, and 12 trials for Color Shapes).

### Secondary outcomes

The secondary outcomes are self-reported quality of life, overall health status, physical activity, perceived stress, depressive symptoms, mindfulness, and sleep quality. We use the EQ-5D-5L survey to measure participants’ health-related quality of life (HRQOL) at baseline, 12, 18, and 24 weeks. The EQ-5D-5 L is easy to administer and is the most widely used instrument to measure HRQOL among individuals with predementia or mild cognitive decline [65]. The EQ-5D-5 L includes both the negative aspects (illness) and the positive aspects (well-being) that are highly associated with older adults’ ability to function independently, mortality rate, and hospitalization occurrence in the long term [66, 67].

The SF-36 Short-form Health Status Questionnaire assesses participants’ overall physical and mental health [68]. The Community Health Activities Model Program for Seniors questionnaire (CHAMPS) assesses participants’ overall physical activity levels [69, 70]. The Mindfulness Attention Awareness Scale (MAAS) and the Five Facet Mindfulness Questionnaire (FFMQ) assess participants’ trait mindfulness levels. These two mindfulness scales are the most common tools applied in physical activity research [71, 72]. The 10-item Perceived Stress Scale (PSS-10) will assess participants’ overall stress levels [73, 74]. The Center for Epidemiological Studies-Depression Scale (CES-D) assesses participants’ depressive symptoms [75]. The Pittsburgh Sleep Quality Index (PSQI;18 items) measures participants’ sleep quality [76]. The 10-meter walk test will assess participants walking speed (meters/sec) over a short distance to determine their functional mobility and gait speed against the age-based normative values [77].

**Device-based measure of daily movement activity.**

This study applied the research-grade activPAL accelerometer (PAL Technologies, Glasgow, UK) to measure physical activity outcomes in both groups during the four measurement bursts that overlap with the mobile cognitive assessment period. ActivPAL is a valid and reliable accelerometer to capture subtle changes in posture (e.g., sitting, standing) and movement (e.g., stepping) among older adults in free-living contexts [78–80]. During each measurement burst, participants wear the activPAL on the thigh for 24 h a day, including sleep time but excluding bathing or swimming time. A copy of the activPAL wearing instructions is provided to participants at their baseline visit.

In the activPAL output file, accumulated steps and minutes of light, moderate, and vigorous physical activity are summarized across all waking hours for each wear day [81]. In this study, the time spent in different intensities of physical activity (i.e., light, moderate, or vigorous) is determined by the activPAL ActivityScore (MET.h) value (a proxy index of energy expenditure levels) with reference to the CDC metabolic equivalents (METs) cutoff values [82]. This device-based data compares physical activity levels (and steps) between the two groups before, during, and after the mindful walking intervention.

### Adherence plan

Participants can decide to drop out of the study at any time without providing a reason, and there are no consequences if they quit the study. A variety of strategies are employed to promote intervention adherence, measurement completion, and study retention. The research team strives to establish a positive rapport with participants and provides responsive support throughout the study period. Prior to enrollment, the staff outlines the potential pros and cons of study participation to let participants make an informed decision about participating and the expectations [83]. The weekly check-in phone calls and text messages conducted during the intervention period also encourage participants to comply with the study protocol. The staff delivers reminder texts or emails to each participant for their upcoming walking sessions and lab visits. During the baseline lab visit, participants should identify two secondary contacts in case the study team can not reach the participant. We offer flexible scheduling to accommodate participants’ time for the walking sessions and visits, including walking on weekend days.

In addition, financial incentives are provided after participants complete the in-person measures and the activity monitoring. Participants receive up to $200 if they complete the entire study with proper compliance (complete all in-lab measures and provide ≥ 70% of the EMA and activPAL data). Specifically, they receive $25 for completing the baseline and the-12 week measures, $60 for completing the 14-day EMA monitoring, $35 for the 18-week measures, and $40 for the 24-week measures. Participants who complete the entire study protocol with ≥ 70% compliance rate are eligible to participate in a drawing to earn an extra $40. Lastly, participants will receive a summary of their physical activity levels during the monitoring period collected by the activPAL device.

### Data preparation, analysis, and management

***Power and sample size consideration***. We conduct apriori power and sample size calculation to detect at least a small between-group effect (Cohen’s *d* = 0.2) based on the available literature showing the anticipated effect sizes from mindfulness-based physical activity programs and cognitive health in older adults [84–86]. The power analysis R package “powerlmm” is used to determine the sample size in the mixed-effects analysis framework with repeated measures. The package identified that a total sample size of 114 participants (57 in each group) would support the detection of the pre-specified small effect size between two groups with sufficient power (≥ 0.80) [87]. We plan for a 15% dropout rate based on the attrition rate in our preliminary study and the annual injury rates (~ 5%) in older adults (i.e., due to serious falls) that lead to unanticipated dropouts [88]. Thus, we will recruit 18 extra participants to account for the attrition, so the actual power for analysis is likely to be greater.

***Statistical analysis***. After the study is completed, we will first examine the distributions of all the quantitative outcome variables prior to any statistical analysis. If any variables violate the normal distribution assumption, we will apply proper sampling distributions and link functions to model the data [89]. We will apply the generalized linear mixed-effects modeling to examine the short- and longer-term effects of mindful walking on cognition and other study outcomes. The repeated measures collected across the study period are the within-subject components, and the group allocation (mindful walking vs. delayed walking group) is the between-subject component in the model. The primary interest in the model is the Time x Group interaction term, which will determine whether significant differences exist in the outcomes between the two groups from baseline to post-intervention. The analyses will be conducted in R program version 4.0.1 [90]. Participants’ baseline characteristics and demographics (e.g., sex, age, BMI, general health status, etc.) will be adjusted in all models. We will also control for participants’ enrollment time of the year (in months) in our analyses to account for any potential seasonal effects that may impact the study outcomes.

***Missing data handling***. The study team minimizes missing data through rigorous staff training before data collection and regular contacts with participants to ensure compliance. We will conduct missing pattern analysis and take relevant measures to reduce missing data-related bias in our analytic models. For example, we will run suggested expectation-maximization imputation methods to compare model results with and without data imputation [91–93]. The analyses of all the study outcomes will be conducted following the intention-to-treat principle [94].

***Data management***. All data collected, including personal information (demographics) from this study will be stored and saved on password-protected and encrypted servers at the university to ensure data safety. No personal information about the screened and enrolled participants will be shared beyond the research team to protect confidentiality. Any published data and articles from this study will be de-identified to exclude any personal information. One graduate-level research assistant is designated to conduct weekly checks of the collected data to promote data quality and identify any errors in data entry, coding, storage, or data uploading.

### Analysis of intervention implementation cost

In addition to testing the intervention efficacy, we will estimate the costs of planning and implementing the described mindful walking intervention using valid methods following the Consolidated Health Economic Evaluation Reporting Standards (CHEERS) 2022 framework [95]. The total program cost is undertaken from two perspectives. First, the payer perspective (the program implementer, but could be a health insurer in the future), and second, the societal perspective. Broadly speaking, the payer perspective includes all costs required to implement the program. We will collect information for all program costs but delineate in our analysis those costs that are solely research related. The research-related costs may not be relevant if the intervention is translated for further roll-out in non-research settings. The societal perspective includes payer costs and participation costs in terms of time and money, such as travel costs to attend the visits and walking sessions.

Specifically, the cost analysis is conducted prospectively with resource use and associated costs captured using micro-costing methods [96]. First, the research team tracks all non-labor costs to implement the intervention. These include, but are not limited to, charges such as transportations borne by the program, activity monitors, supplies, parking, and printing and mailing. Each team member also keeps track of research-specific (e.g., conducting outcome measures) vs. non-research costs (e.g., parking, sending reminders) to delineate costs for future translation into practice. The separation of research and non-research intervention implementation components can increase translation potential.

Second, the team will complete daily logs of time effort throughout the study period to calculate labor costs. Time effort is separated into research and non-research-related categories (e.g., delivering the intervention). This tracked time effort information will be multiplied by staff’s hourly wage/income levels to calculate the estimated labor costs (i.e., unit of time × resource cost/unit of time = labor costs). The labor and non-labor costs comprise the payer perspective (e.g., Blue Cross Blue Shield). To determine the societal costs, we survey participants at baseline and after study completion to understand their participation costs. These costs include, but are not limited to, transportation and time associated with traveling to participate in the study. Given the age of our study population and the walking session protocol, we assume no wages are lost due to retirement or that they are participating outside of work hours or during breaks. Our baseline demographic survey on retirement status and the walking session time logs will determine if our assumption holds. In addition, our participants may have transport costs such as a ride-share, public transport, or other means.

Final cost outcomes will be framed in terms of cost per participant from the payer (or program implementation) perspective and cost per participant from the societal perspective (including participant-borne costs).

***Process measures and program evaluation***. Throughout the study period, the research team will track participants in both groups to document if they attend their scheduled visits for measurement and comply with the EMA (activity and cognition monitoring) protocol. The team will track the walking session attendance and completion rates for the mindful walking group. The number of no-show events, missed sessions, rescheduled sessions, and partially completed sessions will be documented for each participant. Participants in the intervention group will complete a brief evaluation survey during the final lab visit to evaluate and refine this mindful walking study. This survey asks participants to rate program acceptability, usefulness, satisfaction, and overall value of the program using a 7-point Likert scale ranging from 0 (not at all) to 7 (very much).

**Evaluate program recruitment and retention**.

To promote participation and study compliance among AA older adults, our team also quantifies successful strategies for intervention refinement. During the recruitment phase, participants are asked to report how and where they became aware of the program. The team tracks and quantifies monthly enrollment for each recruitment activity. Following the completion of all the mindful walking sessions (after 12 weeks), we will categorize recruitment avenues (e.g., via flyers, emails, word of mouth) and local sites (e.g., churches, senior centers, local partners) and calculate percentages for those screened, not interested, ineligible, and enrolled, respectively. These percentages are calculated among the recruited sample and are broken down by key demographics (e.g., sex, employment, education level). We will compare enrollment yields by avenues and sites using a crosstab table and conduct chi-square test or Fisher’s exact test (if cell frequencies < 5) to determine if any categories of the recruited numbers are significantly higher or lower than the expected values. Identifying the highest- and lowest-yielding recruitment sources, especially for hard-to-reach minority populations, will make considerable contributions to informing recruitment strategies used in future behavioral intervention programs [97–99]. The program retention rate will be calculated post-program (after 12 weeks) and on each follow-up occasion (after 18 and after 24 weeks). The percentages of specific reasons for attrition (e.g., loss to follow-up, no longer interested, medical reasons) will be calculated among the total sample and by demographics. We will further collect the retention rate and reasons for attrition based on the recruitment avenues and sites. The chi-square test (or Fisher’s exact test) again will examine differential attrition rates using a crosstab table.

## Discussion

The preclinical phase leading to the diagnosis of ADRD lasts for years among vulnerable older populations, providing a critical opportunity to deliver risk reduction interventions [100, 101]. This study represents one of the first attempts to evaluate a preventive strategy integrating lifestyle, lower-intensity physical activity, and mindfulness practice to sustain brain health and thus reduce ADRD risk. Findings will generate new knowledge to determine whether this mindful walking program yields efficacy evidence that requires further testing in larger effectiveness trials.

One unique feature of the mindful walking is the integration of physical activity and cognitive activity, which may produce a synergistic effect on cognitive health [102]. Available walking programs for older adults generally focus on reaching the recommended amount of physical activity or steps to obtain health benefits, and thus paying less attention to the “activity of mind” during walking [16, 85, 103]. On the contrary, standard mindfulness programs often restrict (or place less emphasis on) bodily movement (e.g., practicing sitting meditation) to fully engage in mindfulness, so the potential health benefits that can be obtained from physical activity are limited [31, 42, 104]. Mindful walking may be a relatively efficient prevention strategy to promote cognitive health as it simultaneously engages in two modifiable ADRD protective factors - physical activity (walking) and cognitive activity (practicing mindfulness).

Our study design decision to randomize participants to a delayed mindful walking group allows for isolating the efficacy of mindful walking intervention compared to daily routine activities in older adults. There are other meaningful and possible comparators that we plan to carry out in future studies to evaluate the relative impact of mindful walking versus other existing intervention approaches. For example, this mindful walking program can be compared against a brisk walking or a group walking program to understand the role of walking intensity or social interactions during walking. It can also be compared with a seated meditation intervention to disentangle the role of walking or movement on brain health outcomes. Nevertheless, this study is designed to establish the critical first step by evaluating the main effect of mindful walking intervention vs. a usual activity routine control. Study results will provide a foundation for future endeavors to refine and optimize this lifestyle program to protect brain health.

This mindful walking intervention is conducted in the real-life setting (vs. a controlled lab setting) that is considered a priority in contemporary aging research to enhance participant reach and program sustainability [105]. First, we leverage the existing local built environment and utilize a safe walking trail in an urban area to implement this program. The walking trail surrounds a landmark (the South Carolina State House) that is accessible to older adult residents and will attract more attention in the community. Secondly, given evidence showing that exposure to green environments may benefit cognitive health [106, 107], we implement this mindful walking program in outdoor green space to maximize the potential benefits. Lastly, we include mobile-based cognitive assessments to measure sensitive changes in various cognitive domains under naturalistic everyday contexts. Overall, this study optimizes ecological validity (the degree to which the study findings can be applied in real-world situations) in multiple aspects to facilitate future program dissemination and translation in similar settings.

Cost analyses are rare in the behavioral intervention literature, but we track this critical information for future implementation, sustainability and scale-up. This study estimates the time and personnel effort to allow organizations or private institutes to budget the cost required to implement this community-based program [108]. If sufficient evidence suggests the efficacy of this mindful walking on cognitive health and its costs are known, it can be introduced to government agencies or federal health insurance programs and their relevant benefit packages (e.g., State Department of Parks, Recreation and Tourism, Medicare Advantage, and other Medicare plans). Evidence to support payer decisions to adopt such a program in their benefit packages will allow for broader community dissemination. It can also enable the incorporation of this program into existing older adult physical activity programs to promote healthy brain aging and active lifestyles.

The last follow-up assessment is scheduled for 12 weeks post-intervention (24 weeks from baseline) due to constraints in the funding timeline. The duration of this follow-up may not be sufficient to capture the occurrence or diagnosis of neurodegenerative diseases among our participants. However, this study aims to detect program efficacy using sensitive measures of cognitive change or decline to study the between-group differences [57, 63, 109]. This study does not attempt to compare the occurrence of brain diseases or symptoms between the two groups after the intervention. The success of this study will inform future work to extend the follow-up period across years to understand whether mindful walking reduces the likelihood of developing any neurodegenerative diseases among at-risk older adults.

This mindful walking program requires less formal instruction and fewer resources than traditional mind-body interventions and therapies, such as yoga, qigong, and tai chi [110]. It also does not require a walking partner or the group arrangement - a common feature of existing walking or mindfulness-based programs for older adults [85, 111]. The individual-based activity in this program facilitates the arousal of a mindfulness state and represents a valuable feature when social distancing is recommended. Although this program is restricted to a single walking site and each session is supervised by the research staff, these arrangements are meant to ensure the quality of program fidelity and outcome measures, especially in an early-stage study to rigorously evaluate program efficacy. This close supervision does not need to continue when the intervention is adopted later outside of the research context.

Most importantly, this study has the potential to address gaps in the literature about what interventions are most suited to narrow ADRD disparities in the AA older adult populations. This program also does not place strict inclusion criteria regarding physical functioning, allowing it to include more diverse older adult populations to enhance generalizability or external validity. For example, AA older adults who may not be eligible to join existing physical activity programs due to limited mobility are still eligible to participate in this program using a walking tool (e.g., cane, walker). We expect this study can contribute to the diversity, equality, and inclusion of ADRD prevention work by improving the representation of underserved older adults in the greatest need.

## Conclusion

This study describes the study protocol of a lifestyle behavior intervention designed to sustain cognitive health in AA older adults. This program is implemented in a less controlled local infrastructure to facilitate long-term behavior change in a real-life environment. Supportive findings from future large-scale trials can inform a preventive strategy that is more sustainable and can be performed individually and repeatedly in daily life. The success of this mindful walking program also holds promise as a potentially effective and affordable strategy for promoting active lifestyles in older populations, making itmore likely to be sustainable in the long run.

### Trial status

The recruitment is currently ongoing at the time of this study protocol submission. The study enrollment will continue until the end of 2024. The content reported in this study reflects the first version of the trial protocol registered on ClinicalTrials.gov (NCT06085196).

### Electronic supplementary material

Below is the link to the electronic supplementary material.

Supplementary Material 1

## Data Availability

The final data used and/or analyzed in the current study are available from the corresponding author upon reasonable request.

## Abbreviations

ADRD: Alzheimer’s disease and related dementias
AA: African American
CAB: Community advisory board
OSA: Office for the Study of Aging
MoCA: Montreal Cognitive Assessment
CERAD: Consortium to Establish a Registry for Alzheimer’s Disease subjective cognitive decline scale
EQ-5D-5L: EuroQol 5 dimensions and 5 levels of response quality of life survey
CHAMPS: Community Health Activities Model Program for Seniors physical activity questionnaire
FFMQ: Five Facet Mindfulness Questionnaire
MAAS: Mindfulness Attention Awareness Scale
SF-36: 36-Item Short Form Health Survey
PSS-10: 10-item Perceived Stress Scale
CES-D: Center for Epidemiological Studies-Depression Scale
PSQI: Pittsburgh Sleep Quality Index
IADL: Instrumental Activities of Daily Living Scale
PSSS: perceived social support scale
CHEERS: Consolidated Health Economic Evaluation Reporting Standards
ISC: University of South Carolina
